# Analysis of Bulk Sample of Salicylic Acid by Application of Hydrotropic Solubilization Method

**DOI:** 10.4103/0250-474X.49135

**Published:** 2008

**Authors:** R. K. Maheshwari, V. Chavada, S. Varghese, K. Shahoo

**Affiliations:** Department of Pharmacy, Shri G.S. Institute of Technology and Science, 23, Park Road, Indore-452 003, India

**Keywords:** Salicylic acid, hydrotropy, sodium salicylate, ibuprofen sodium, titrimetry

## Abstract

In the present investigation, the poorly water-soluble drug, salicylic acid has been solubilized using 0.5 M ibuprofen sodium and 2.0 M sodium salicylate solution as hydrotropic agents for the titrimetric analysis precluding the use of organic solvents. Both hydrotropes are economic and pollution-free. The mean percent estimation of salicylic acid estimated in bulk sample by Indian Pharmacopoeial method is 98.78%. The mean percent estimation by ibuprofen sodium method and sodium salicylate method are 99.25% and 98.82%, respectively. The results of analysis by the proposed method are very close to the results of analysis by the standard method. This confirms the accuracy of the proposed method. The proposed method was validated statistically by low values of statistical parameters viz. standard deviation, percent coefficient of variation and standard error. The proposed method is new, accurate, simple and economic.

Hydrotropic solubilization technique is one of the methods used to enhance the aqueous solubility of insoluble or slightly soluble drugs. This technique involves the addition of large amount of additives in presence of which the aqueous solubility of the solute shows multifold enhancement^1^–^17^. Maheshwari analyzed various poorly water-soluble drugs, using hydrotropic solubilization phenomenon viz. frusemide^1^, tinidazole^2^, ketoprofen^3^, cefixime^4^ and ketoprofen^5^. Maheshwari *et al*. have developed various analytical techniques using hydrotropic solubilization phenomenon to analyze poorly water-soluble drugs, aceclofenac^6^, hydrochlorothiazide^7^, cephalexin^8^ and piroxicam^9^. The Indian Pharmacopoeial method of titrimetric analysis of salicylic acid uses an organic solvent, ethanol for the solubilization of the drug. Drawbacks of using an organic solvent include toxicity, high cost and environmental hazards. The primary objective of this study was to preclude the use of organic solvent and to employ hydrotropic solubilizing agents, ibuprofen sodium and sodium salicylate, which are economic.

All chemicals and solvents used were of analytical grade. Salicylic acid (S. D. Fine Chemicals Limited, Mumbai) was procured from market. Ibuprofen was obtained as gift sample from Shree Pharmaceuticals Ltd., Indore, India.

Solubility of salicylic acid was determined in distilled water and different concentrated solutions of hydrotropic agents at 27±1°. Enhancement of solubility of salicylic acid in 0.5 M ibuprofen sodium solution and 2.0 M sodium salicylate solution was more than 12-folds and 6- folds respectively (as compared to solubility in distilled water).

For the preparation of 0.5 M ibuprofen sodium solution, 10 g sodium hydroxide was dissolved in 200 ml distilled water. Ibuprofen (51.6 g) was added little at a time and stirred continuously to dissolve. After complete addition of ibuprofen, the pH was adjusted between 7.5-8.0 with additional sodium hydroxide solution to assure complete neutralization of ibuprofen.

For the analysis of salicylic acid by Indian Pharmacopoeial method (IPM)^18^, accurately weighed (0.3 g) salicylic acid bulk sample was solubilized in 50 ml of ethanol. After adding 20 ml distilled water, it was titrated with sodium hydroxide solution (0.1 M) using phenol red solution as indicator. Necessary blank determination was adjusted to get drug content (Table 1).

**TABLE 1:** ANALYSIS DATA OF SALICYLIC ACID BULK DRUG SAMPLE WITH STATISTICAL EVALUATION

Method	Mean % estimation	Standard deviation	% Coefficient of variation	Standard error
IPM.	98.78	1.630	1.650	0.815
ISM	99.25	1.069	1.077	0.534
SSM	98.82	0.627	0.634	0.313

IPM refers to Indian Pharmacopoeial method, ISM refers to ibuprofen sodium method and SSM refers to sodium salicylate method; (n=4).

For the analysis of salicylic acid by ibuprofen sodium method (ISM), accurately weighed (0.3 g) salicylic acid bulk sample was solubilized in 40 ml of ibuprofen sodium solution (0.5 M) in a conical flask by shaking for about 5 min and titrated with sodium hydroxide solution (0.1 M) using phenol red solution as indicator. Necessary correction was done by conducting blank determination and amount of salicylic acid was calculated (Table 1).

For the analysis of salicylic acid by sodium salicylate method (SSM), accurately weighed (0.3 g) salicylic acid bulk sample was solubilized in 50 ml of sodium salicylate solution (2.0 M) in a conical flask by shaking for about 5 min and titrated with sodium hydroxide solution (0.1 M) using phenol red solution as indicator. Necessary corrections were done by conducting blank determinations and amount of salicylic acid was calculated (Table 1).

As evident from (Table 1), the mean percent estimation of salicylic acid estimated in bulk sample by IPM is 98.78%. The mean percent estimation by ISM and SSM are 99.25% and 98.82%, respectively. The results of analysis by the proposed method are very close to the results of analysis by standard IPM. This confirms the accuracy of the proposed method. The proposed method is validated statistically by low values of standard deviation, percent coefficient of variation and standard error (Table 1). Like salicylic acid, other poorly water-soluble drugs can also be subjected to analysis by using hydrotropic solubilization method. It is, thus, concluded that the proposed method is simple, cost-effective, accurate, safe and precise and can be successfully employed in the routine analysis of salicylic acid in bulk drug sample. There is a good scope for other poorly water-soluble drugs which may be tried to get solubilized by suitable hydrotropic agents to carry out their titrimetric analysis excluding the use of costlier and unsafe organic solvents.
